# Drag of Clean and Fouled Net Panels – Measurements and Parameterization of Fouling

**DOI:** 10.1371/journal.pone.0131051

**Published:** 2015-07-07

**Authors:** Lars Christian Gansel, David R. Plew, Per Christian Endresen, Anna Ivanova Olsen, Ekrem Misimi, Jana Guenther, Østen Jensen

**Affiliations:** 1 Norwegian University of Science and Technology, Department of Marine Technology, Otto Nielsens vei 10, Trondheim, Norway; 2 SINTEF Fisheries and Aquaculture, Department of Aquaculture Technology, Brattørkaia 17C, Trondheim, Norway; 3 NIWA, Riccarton, Christchurch, New Zealand; 4 Den Norske Veritas, Professor Brochs Gate 2, Trondheim, Norway; 5 Statoil, Strandvegen 4, Stjørdal, Norway; University of California San Diego, UNITED STATES

## Abstract

Biofouling is a serious problem in marine aquaculture and it has a number of negative impacts including increased forces on aquaculture structures and reduced water exchange across nets. This in turn affects the behavior of fish cages in waves and currents and has an impact on the water volume and quality inside net pens. Even though these negative effects are acknowledged by the research community and governmental institutions, there is limited knowledge about fouling related effects on the flow past nets, and more detailed investigations distinguishing between different fouling types have been called for. This study evaluates the effect of hydroids, an important fouling organism in Norwegian aquaculture, on the forces acting on net panels. Drag forces on clean and fouled nets were measured in a flume tank, and net solidity including effect of fouling were determined using image analysis. The relationship between net solidity and drag was assessed, and it was found that a solidity increase due to hydroids caused less additional drag than a similar increase caused by change in clean net parameters. For solidities tested in this study, the difference in drag force increase could be as high as 43% between fouled and clean nets with same solidity. The relationship between solidity and drag force is well described by exponential functions for clean as well as for fouled nets. A method is proposed to parameterize the effect of fouling in terms of an increase in net solidity. This allows existing numerical methods developed for clean nets to be used to model the effects of biofouling on nets. Measurements with other types of fouling can be added to build a database on effects of the accumulation of different fouling organisms on aquaculture nets.

## Introduction

Biofouling, the accumulation of marine organisms on surfaces, is a serious problem in marine aquaculture. The occlusion of mesh openings due to biofouling has a number of negative effects, including increasing drag forces on fish cages and restricting the water exchange through the nets ([1], [2], [3], [4]). In finfish aquaculture, net cages change their shape by deflection and deformation, depending on current velocity, original shape, construction of the cage, type and state of netting as well as weight configuration (sinkertube or lump weights) ([5], [6]). The increase of drag caused by biofouling can introduce significant additional deformation of net cages ([4], [6]), which in turn will lead to an increased risk of contact between the net and weight system. Chafing on the net from the chain supporting the sinker tube has been the cause of a number of escape incidents, some of which have been relatively large [7]. Technical standards, such as the Norwegian Standard NS9415 [8], regulating the use of structural elements of fish cages and farms, acknowledge an effect of the accumulation of biofouling on hydrodynamic loads on fish cages, but they fail to describe the effect of fouling in detail. The accumulation of biofouling has technical relevance, but it also affects fish welfare. Fouling related cage volume reduction (due to deformation of the net cage) increases the actual stocking density, and reduced water exchange across nets caused by the accumulation of fouling reduces waste removal from and oxygen supply into fish cages [1].

Milne [9] investigated hydrodynamic forces on clean and fouled nets at various current velocities and demonstrated that these forces can be up to 12.5 times higher on fouled than on cleaned nets. Swift et al. [4] measured the effect of biofouling, mainly consisting of skeleton shrimp *Caprella* sp., on the drag forces on 1 m^2^ net panels using a bridle-pulley-load cell configuration in the field, and demonstrated that drag of fouled nets may be over 3 times that of clean nets. Several other authors also report fouling related drag increase (e.g. [10], [11], [12]), which underlines the potential for biofouling accumulation to significantly increase forces acting on net panels.

Numerical models exist to predict the behavior of clean nets and fish cages in waves and currents, but models that accurately take the effects of biofouling into account do not exist. This may be due to a lack of knowledge on the effect of biofouling buildup on the hydrodynamic properties of fish nets and cages. Most studies investigating the effect of fouling on the forces on nets do not distinguish between different fouling types even though different types of fouling will affect drag forces differently. Swift et al. [4] highlight the importance of quantifying the effects of biofouling on hydrodynamic properties of nets for the design of aquaculture net pen systems and they suggest a separation of fouling types in future studies. In Norway, the world’s leading aquaculture producer of salmonids [13], the hydroid *Ectopleura larynx*, the mussel *Mytilus edulis* and algae are among the main fouling organisms on salmon cage nets [14]. In particular, the hydroid *E*. *larynx* dominates fouling communities on coastal fish farms in Southwest- and Mid-Norway during the peak of the fouling season between July and November [15]. Given the large biomass of hydroids on aquaculture nets in Norway, hydroids were chosen as a model organism in the present paper.

This study aimed to i) investigate the change in drag forces acting on net panels with different solidities and various levels of biofouling and ii) establish a method to parameterize biofouling to enable predictions of the effects of biofouling on net drag using existing numerical models. Measurements were made of the drag on clean nets with a range of solidities, and on initially clean nets of two solidities on which different amounts of hydroids were allowed to grow. The amount of biofouling was measured by the change in solidity, as assessed by image analysis of photographs of the net panels. The relationships between drag and solidity were compared for the clean and fouled nets, and these relationships were used to develop a parameterization for hydroid fouling in terms of an equivalent increase in the clean net solidity.

## Materials and Methods

The work presented in this article includes tests on nets fouled with hydroids and ascidians. These organisms are not included in the Norwegian regulations for animal research and no ethical approval is needed for tests on these in Norway. Regardless, the Regulation on Animal Experimentation was followed to great extent. More information on the Norwegian regulation is found at http://lovdata.no/dokument/SF/forskrift/1996-01-15-23#Kapittel_2 and at the webpages of the Norwegian Forsøksdyrutvalget (www.fdu.no). The fish farm at which net panels were deployed is a research farm site licenced by the Norwegian Directorate of Fisheries. Permission for the deployment of clean nets at the farm site was given by the licence holders for and the operators of the fish farm, namely ACE Aquaculture Engineering and Salmar AS. No further permission was required to conduct the tests. The tests did not include endangered or protected species.

### Setup and procedure

**Fig 1:**
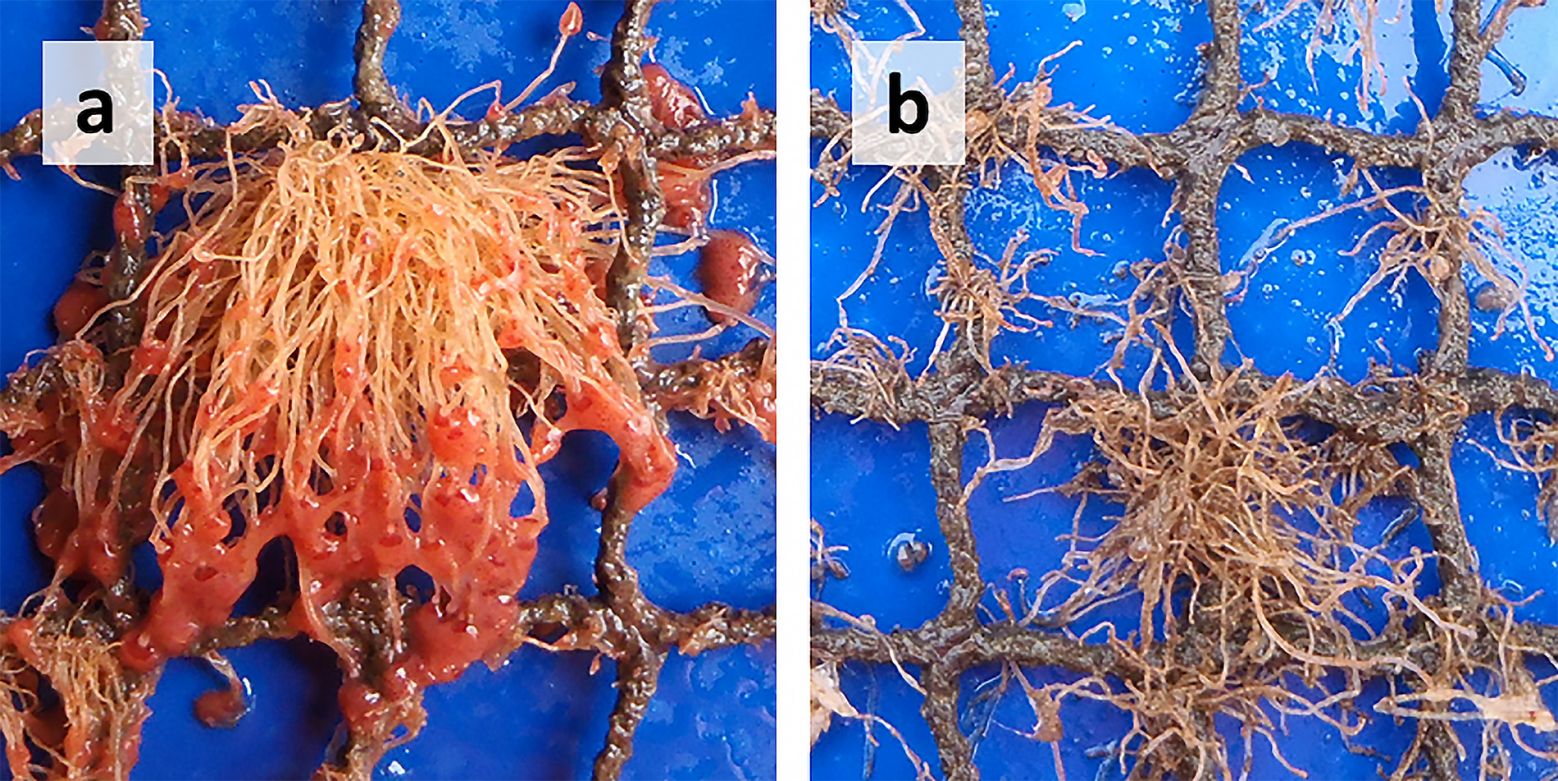
Hydroid fouling on aquaculture nets. The images show net panels on the same fish farm, but at different times with approximately one month between the images. Hydroids found on Norwegian salmon cages have bright pink hydranths (a), but on some nets, probably mostly in late autumn and early winter, hydranths may be lost or retracted (b).

The forces on fouled and clean nets were measured in a low-turbulence recirculation flume tank. The tank was 13 m long, 0.6 m wide and 0.5 m deep. The biofouling on nets consisted mostly of hydroids (see also section *'Clean nets and accumulation of biofouling on net panels*') and the water in the flume tank was freshwater. In a pre-test, most hydroids lost their hydranths within seconds after their submergence in freshwater, which may result in changes in the drag force of fouled nets. The hydranths are the pink parts on top of the stems of hydroids (hydrocaulus) in Fig 1a. Therefore, all nets were kept in freshwater for a few minutes to remove the hydranths from the hydroids before they were mounted in the flume tank. Only the hydranths were lost prior to the measurements, but the hydrocauli of hydroids stayed intact. Hydroids are found in different developmental stages on Norwegian aquaculture nets. Two examples from hydroid-fouling on coated salmon nets samples that were submerged at a commercial Atlantic salmon farm in southern Norway are shown in Fig 1. The picture in Fig 1a was taken in August 2011 and here the hydroids have bright pink hydranths. Most hydroids in Fig 1b, taken in late October 2011, do not have hydranths. Pyefinch and Downing [16] found that hydroids may lose and re-grow hydranths in the sea. The results from this study describe effects of net fouling with hydroids in a state similar to that shown in Fig 1b. However, they may also help to estimate effects of any filamentous, flexible fouling in the hydroid size range (the length scale is centimeters and the diameter scale is millimeters).

**Fig 2:**
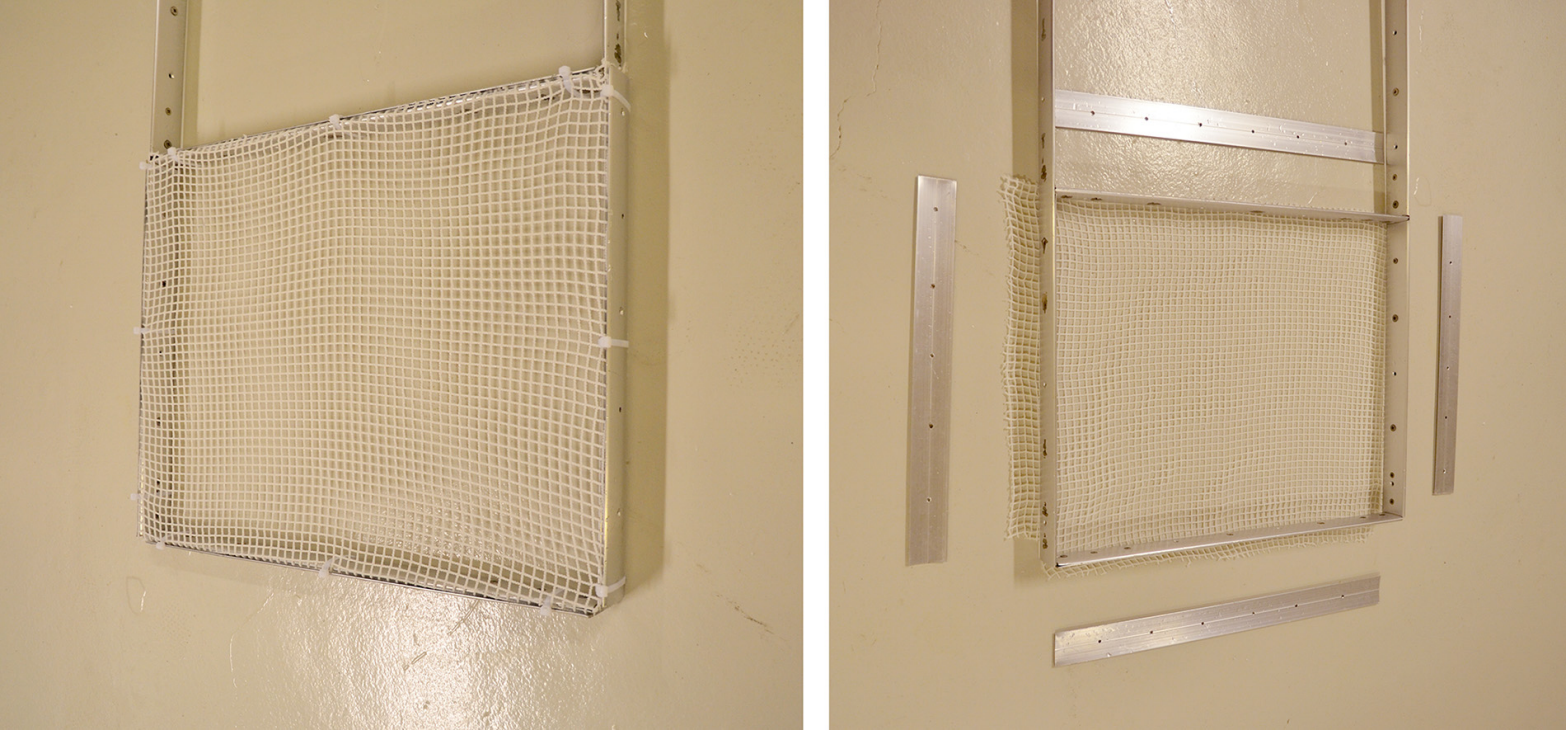
Frame used in all tests. The frame was made from curved aluminum profiles. The same profiles were used to clamp the nets to the frame in order to minimize drag on the mounting system.

All nets were mounted in a frame by clamping the netting in between two metal profiles on all four sides of the frame as shown in Fig 2. The nets were stretched over the frame and clamped under tension in order to suppress net deformation during the experiments. To clamp nets under tension the net panels had to have initial sizes bigger than the frame size and any parts sticking out from the back of the frame were trimmed off after mounting the nets. Two techniques for the setup for drag measurements of net panels are available: i) the net is placed in a tank large enough to avoid any wall effects, that is the flow around the panel does not feel the wall or ii) the net spans almost the entire cross section of the tank, thus assuring almost all water to be pressed through the net. Both techniques have advantages and disadvantages. When nets are placed in a large tank to avoid wall effects, the blockage caused by nets will force water around the net panel, thereby changing angle between the water flow direction and the net panel. A directional change of the water flow will affect the drag on the net. Flow attenuation and re-direction will be dependent on the blockage caused by the net, which changes with solidity. Flow re-direction around the net panel further leads to a change in the pressure distribution and thus a change in the drag force. Even when the change of the flow field around net panels can be investigated, it will differ for nets causing different blockage and thus, comparing the drag on different nets gains additional uncertainty. For these reasons we decided for a setup where the nets span almost the entire cross section of the flume tank. The downside of such a setup is that the flow through and around the net will interact with the walls of the tank. However, with little spacing between the net and the tank walls, almost all the water will have to pass the net and wall effects are related to solidity. This study compares the drag on different net types and we assume that there is a direct relationship between net solidity and drag. As long as any effect on the drag measured on the nets is proportional to the net solidity, a comparison of nets based on drag will be valid.

**Fig 3:**
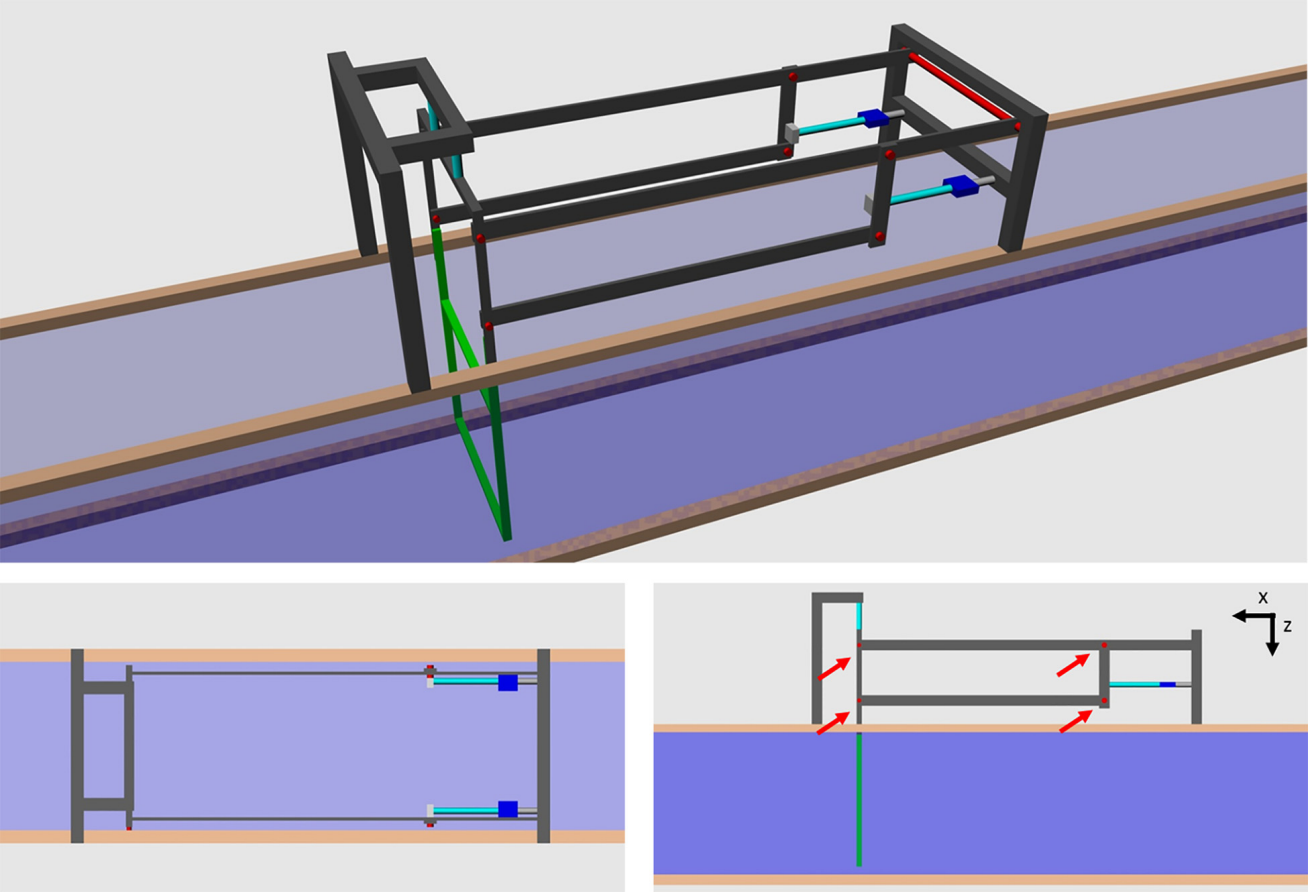
Sketch of the measurement system. The frame in which nets were mounted is green, springs are indicated in turquois, pivot points are red (and marked with red arrows) and strain gauges are the blue boxes.

The frame was 0.5 m wide and 0.4 m high and it was centered in the x- and z-direction (Fig 2). The distance between the walls of the flume tank and the frame was 0.05 m on all sides. The frame was made from thin, oval aluminum profiles for minimal resistance, and it was connected to a mounting frame as shown in Fig 3. Due to the pivot points indicated in the figure the frame could move in the x- and z-directions, but only the forces in x-direction are presented in this study. Although the setup allowed a slight change in angle (tilt) of the frame, these changes were in the range of one degree. Effects on the measured forces due to tilting of the frame were therefore neglected.

The forces on all nets were measured at a flow speed of 0.1 ms^-1^. All nets were photographed under water in the flow and the solidity was determined from the images as described below in the section *Image acquisition and analysis*.

Visualisation of the flow around single twines of clean nets was performed using dye released from a hollow needle about 10 cm upstream from the nets.

Data were collected on two occasions. In the first set of experiments (2009), both clean and fouled nets were tested. Additional measurements of the drag on clean nets were conducted in 2011 to increase the number of data points. The same experimental apparatus was used for both experiments.

### Image acquisition and analysis

The net solidity (S_n_) is expressed as the ratio of the solid fraction of the net to the total net outline area, and it is measured from images:

$$S_{n}=\frac{solid\text{​ }area}{total\;net\;outline\;area}$$ (1)

To determine the net solidity, the nets were photographed with a NIKON D7000 SLR camera (Nikon, Chiyoda, Tokyo, Japan), with a 18–105 mm lens. The camera was placed in a NIMAR (NIMAR, Florida, USA) underwater housing.

**Fig 4:**
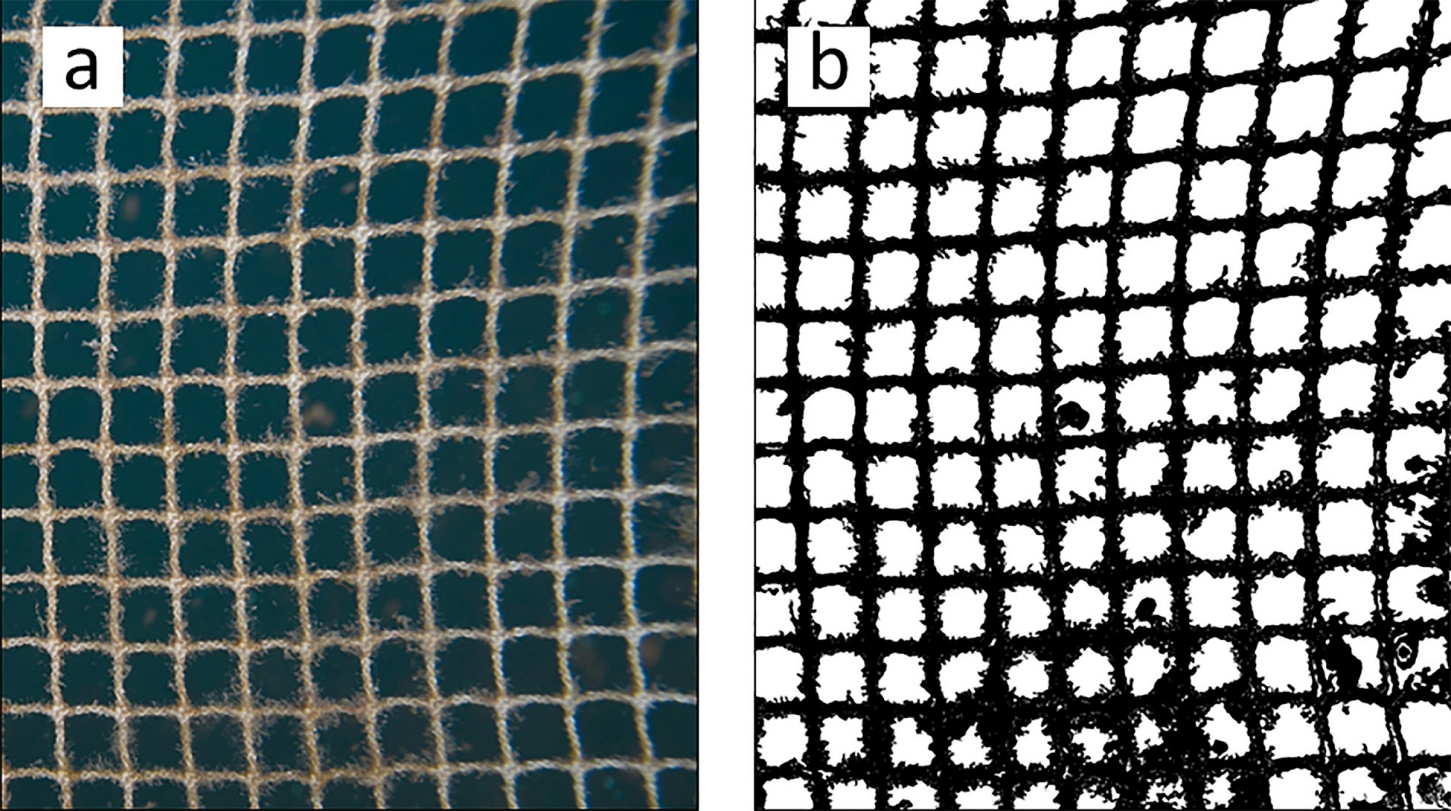
Example for the image analysis. (a) the original (RGB) image and (b) the segmented (binary) image as a result of the image analysis. The net solidity can be determined from the ratio of black (net and fouling) and white pixels (background).

Digital images were taken of all nets stretched on the frame in the current. The analysis is similar to that used by [15] with some differences. The RGB (Red, Green, Blue) images from the camera were preprocessed before segmentation so that they could be analyzed for net solidity. RGB images (Fig 4a) were converted to HSL (Hue, Saturation, Luminance) color space. The Luminance channel image was used for segmentation. Given the large variation in background illumination, contrast, and sharpness in the images, it was necessary to use manual thresholding for segmentation. From the segmented binary images (Fig 4b) (black and white pixels), the net solidity was determined by comparing the amount of pixels belonging to the background (net openings) and the net and fouling as described in [15].

### Clean nets and accumulation of biofouling on net panels

Nine nylon nets with solidities between 0.18 and 0.43 were used in this study. The characteristics of all nets are summarized in Table 1.

**Table 1:** Characteristics of the clean nets used in this study. The nets used for tests with biofouling were N1 and N2. Solidity is calculated by image analysis.

Net number	Solidity [Sn]	Twine diameter [mm]	Net aperture length [mm]
N1	0.2	2.61	23.5
N2	0.29	2.45	13.6
N3	0.36	1.33	6.35
N4	0.43	1.3	4.25
N5	0.18	2.38	24.46
N6	0.24	2.91	22.6
N7	0.32	2.07	11.67
N8	0.26	1.84	13.27
N9	0.29	2.74	20.97

Uncoated nylon net panels (60 × 60 cm) of two mesh sizes (13.6 mm and 23.5 mm net aperture length) were attached to PVC frames and deployed at 5 m depth in the proximity of cages at a commercial Atlantic salmon farm located at Ørnøya (63.7517° N, 8.4353° E), Mid-Norway, in August 2009. These nets were identical to net panels N1 and N2 in Table 1. After two weeks, a macro-fouling community consisting of mainly the hydroid *Ectopleura larynx* had established on most net panels. All nets that were fouled with macro-fouling other than hydroids were dismissed from the study. To be able to measure drag forces on these net panels with varying amounts of biofouling attached to them, 3 net panels of each mesh size were removed after 2, 5 and 10 weeks of immersion. Net panels were transported submerged in seawater to the laboratory and temporarily kept in recirculating water at 12°C.

### Force and flow measurements

Two load cells were used to measure the in-line (drag) forces on the models. The output from the load cells (AEP Transducers, model: TCA.315.R3, Code: CTCA5K5) were amplified and transferred to a PC via a HP3852 data acquisition system (using HP44726A multiplexers). The repeatability of the load cells was ≤ ± 0.01% and linearity and hysteresis was ≤ ± 0.03%. The sampling was conducted for 240 seconds at 50Hz during all tests. The flow speed was set to 0.1 ms^-1^ using a NORTEK Vectrino for control measurements 1 m upstream from the net panels in the center of the tank. The same instrument was used to confirm this flow speed several times before and after tests. Different solidities of nets did not affect the flow speed. The time period of the measurements assured that half the tank's volume passed the net panels during each individual test. Since the water flow was started at least an hour before the first tests were conducted, large variations in flow along the length of the tank are unlikely and no large variations were present during flow measurements. Small irregularities of the flow speed on time or length scales much smaller than the measurement period and the length of the water volume passing the nets during each test did not distinctly influence the measurement results, as all data was time averaged. A low pass filter (15 Hz) was used to minimize noise from the 50 Hz electrical supply network. The strain gauges were calibrated using a set of known weights so that voltage output [V] could be expressed as a force [N].

### Data processing and statistics

The time series from both strain gauges were filtered separately by calculating the time average of and the standard deviation within the time series and then dismissing all data points that were outside the range of 3 times the standard deviation around the time average, that means that under, the assumption of normal distribution of data, roughly 0.3% were removed [17]. This was done in order to discard peaks in the force measurements that may have been caused by variations in the voltage from the power supply of the PC caused by variations of the electrical network. The time-averaged total force was then calculated as the sum of the mean values of the filtered time series from both strain gauges. The forces on the bare frame were subtracted from the forces obtained from tests with net samples.

The following data were analyzed: 1) Drag on clean nets with different solidities, 2) Drag on nets (type N1 in Table 1) with different amounts of hydroid fouling and 3) Drag on nets (type N2 in Table 1) with different amounts of hydroid fouling. The data was analyzed to ascertain if the data sets can be treated as independent (that is, if entries in the different data sets are likely to be from distributions with different means) and if there are differences in the relationship between net solidity and drag force between the data sets. As will be shown, an exponential function described the relationship between drag and solidity reasonably well (correlation for clean nets between solidity and drag: R^2^ = 0.96):

$$F_{d}=ae^{bS_{n}}$$ (2)

where F_d_ is drag force and S_n_ is net solidity. The deviations of measured values (the residuals) from the exponential model for clean and fouled nets were tested for normal distribution using Lilliefors-test with the outcome that the assumption of normal distribution of the data could not be rejected (5% significance level) and homogeneity was given (Levene's test, 5% significance level). These results justify the use of ANOVA (Analysis of Variance) to study differences between the data series specified above.

For further analysis the lognormal transformation was applied to the data. These transformed samples are used to estimate the parameters of the linear regression equation:

Y=A+bx (3)

Where *Y* = ln(*F*_*d*_) and *A* = ln(*a*).

The method of least squares was applied to estimate the parameters of Eq (3) [18] and the variances of parameter estimators for A and b, the unbiased estimate of the model error variance were calculated. Parameters A and b were assumed to be normally distributed with mean values estimated by the least squares method. ANOVA was used to study how different the data series on clean and fouled nets are.

In ANOVA the following hypothesis was tested:

- *H*_0_: *μ*_1_ = *μ*_2_ = *μ*_3_ –means of regression parameters for all samples (data series) are the same,
- *H*_1_: at least two of the means are not equal,
- *α =* 0.05 significance level.

The method calculates the ratio f of the variance between the data series versus within the data series. This ratio f is assumed to be a value of the random variable F distributed following the F-distribution with corresponding degrees of freedom. If probability P that f is in the upper right tail of F-distribution is more than significance level α then the null hypothesis $H_{0}$ is true. That means there is evidence that those samples are most likely from a distribution with the same mean value.

Forces on clean nets were measured in 2009 and 2011. Settings on the measurement system were different between 2009 and 2011 measurements, but nets that were included in both measurement periods could be used to find a calibration factor between 2009 and 2011 measurements.

Drag forces are expressed either dimensionless by normalizing results to a reference net or as a drag coefficient C_d_

$$C_{d}=\frac{2F_{d}}{\rho u^{2}A}$$ (4)

where F_d_ is the drag force, ρ is the water density, u is the flow speed and A is the outline area of nets.

## Results

**Fig 5:**
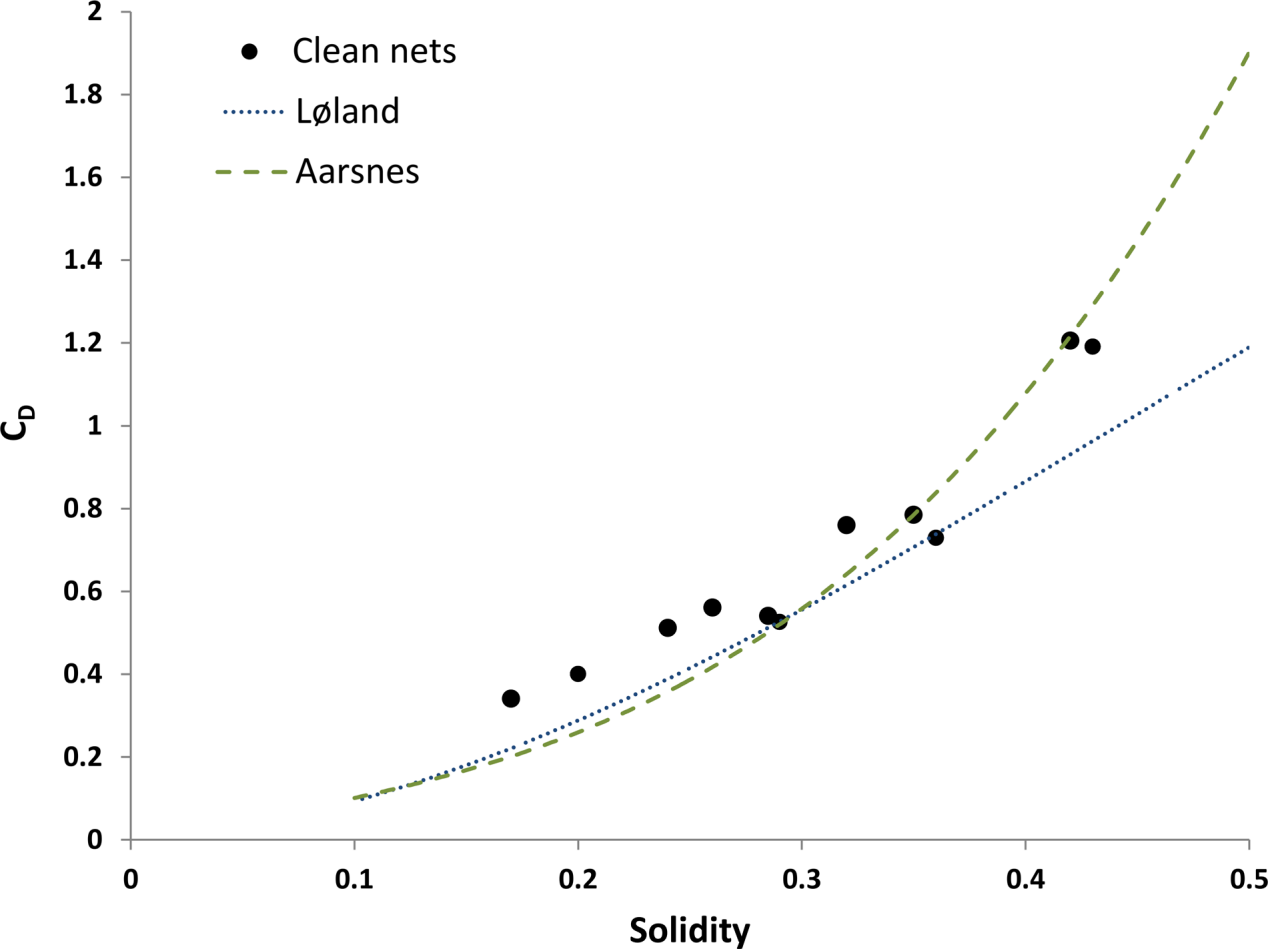
Drag coefficient of clean nets. The broken lines are drag formulations by [19] and [20].

Fig 5 shows the drag coefficients (based on the outline area of nets) of all clean nets together with drag coefficients predicted by formulations by Aarsnes [19] and Løland [20]. Drag coefficients of net panels found in this study are within the range of those predicted by existing empirical and semi-empirical formulations, including nets with solidities exceeding the solidity range these formulations are validated for (for example, [20] based his formulation on data on nets within a solidity range from 0.13 to 0.32). Up to solidities of about Sn = 0.35 both formulations describe the present data well. The drag coefficient of the most solid net in this study (Sn > 0.4) fits well to the C_d_ predicted by Aarsnes [19], while Løland [20] predicts lower drag coefficient.

**Fig 6:**
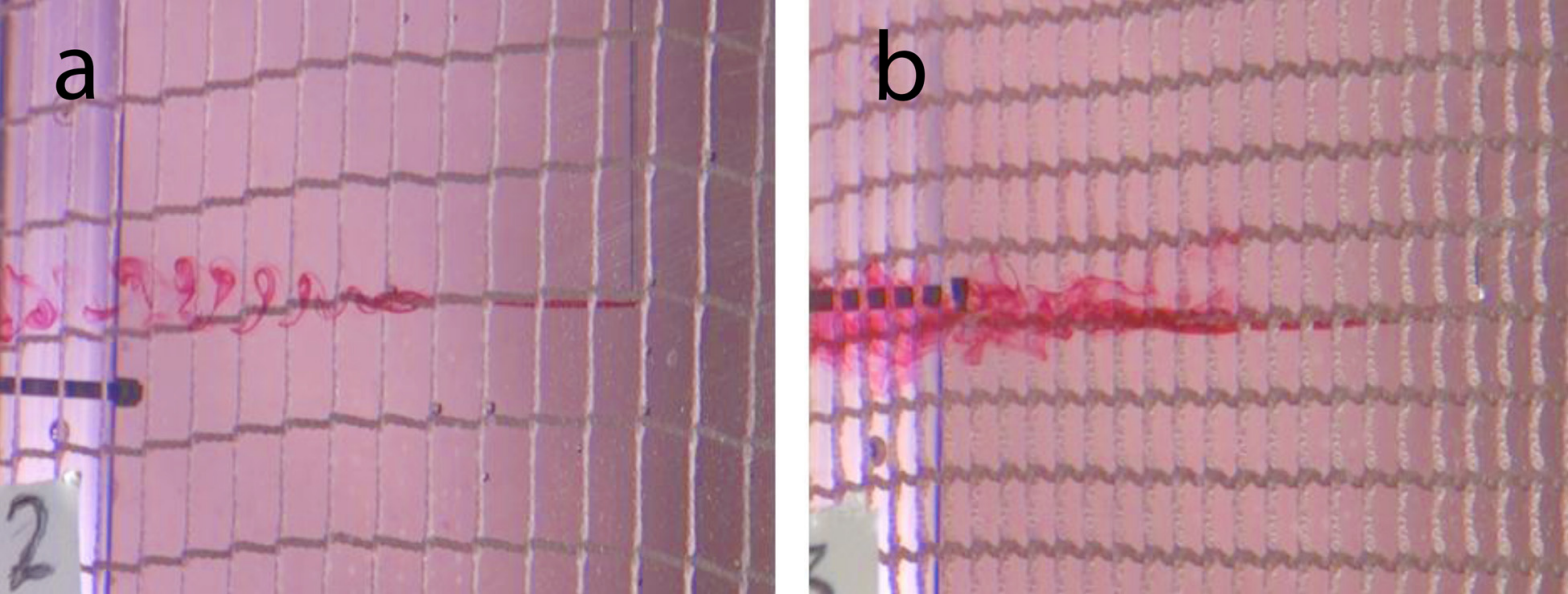
Visualization of the flow past net twines of different nets. The solidities of the nets were (a) 0.18 and (b) 0.32. Dye was released at the height of a net twine, mid-way between the intersection of twines.

**Fig 7:**
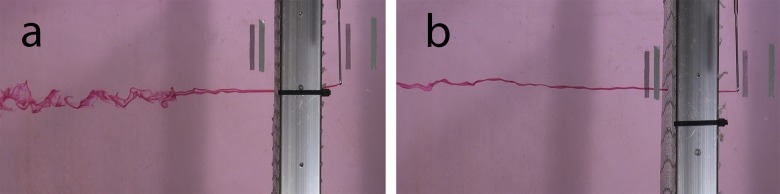
Flow through nets. Examples of flow through net apertures of nylon nets with different twine diameters and net aperture sizes. The solidities of the nets were (a) 0.18 and (b) 0.32. Dye was released in line with the center of a net opening.

Dye experiments revealed high variability in the wake structure downstream from strands of different types of clean nets. Fig 6 shows two examples of dye spreading in the wake of net twines. The flow speed was approximately 0.05 ms^-1^ and the twine thicknesses were roughly 2.5 mm, giving a Reynolds number of 125. The flow past twines of net N7 (Fig 6a) forms a vortex street similar to the flow that is expected around circular cylinders at similar Reynolds numbers, while the flow around a twine of net N5 (Fig 6b) is less organized and does not result in the shedding of big vortices. Different wake structures downstream from nets were also evident from dye dispersion tests downstream from net openings. Fig 7 shows that the flow downstream from net openings becomes unstable at different distances for different nets.

**Fig 8:**
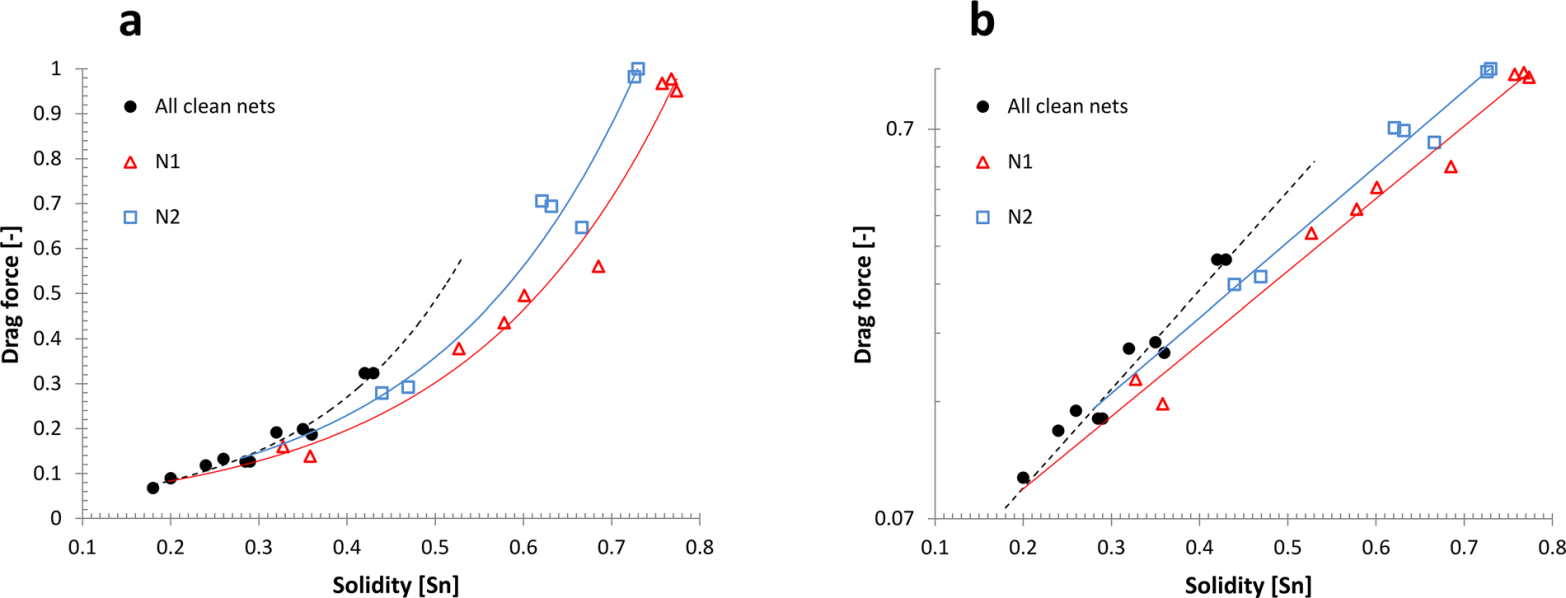
Drag on and solidities of clean and fouled nets with drag on a normal (a) and logarithmic (b) y-axis. Drag forces are normalized to the net with the highest drag (the most solid fouled net of type N2) to ease comparisons between the nets. All data points are averages with standard deviations ranging from 0.0175 to 0.088.

Fig 8 shows drag forces and solidities of clean and fouled nets with drag on a normal (a) and logarithmic (b) y-axis. Drag forces have been normalized by dividing by the drag of the nets with the highest drag (of the most solid fouled net of type N2) to ease comparisons between the nets. All data points are averages with standard deviations ranging from 0.0175 to 0.088. The drag force experienced by a net panel increases with increasing net solidity and exponential fits describe the relationship between S_n_ and drag force well for all data sets (R^2^ = 0.96, 0.969 and 0.978 for clean nets, fouled nets N1 and fouled nets N2, respectively). The interception of exponential fits based on clean and fouled net data is at Sn = 0.2 (N1) and Sn = 0.28 (N2).

All three data sets are likely to be from distributions with different mean values (F_2.297_ = 202.88; p << 0.05). Parameter *b* of the lognormal transformed data was significantly different between data from clean and fouled nets (F_2.297_ = 749.75; p << 0.05) and also between the two fouled net data sets (F_1.198_ = 159.21; p << 0.05). That means the inclination of linear fits to lognormal transformed data is different between the data sets for clean and fouled nets. The inclination of the best exponential fit to the clean net data plotted with log-normal axes is very different from those of the best exponential fits to the fouled net data sets, but the inclination of the two fouled net data sets differs only slightly (y = 0.218*exp(5.9069x), y = 0.2999*exp(4.2888x), and y = 0.3259*exp(4.4666x) for clean nets, fouled nets type N1 and fouled nets type N2, respectively). According to these exponential fits, an increase of solidity by 0.3 increases drag of clean nets by a factor of 6, while the same solidity increase almost quadruples the drag of fouled net fits. The drag between clean nets and the fouled nets than differs by far beyond 100%, while the difference is just above 10% between the fouled net types.

## Discussion

### Solidity as measure for fouling

Solidity for clean and fouled nets was measured using image analysis which is a very accurate method (see [15] for a detailed description), but it should be noted that the method does not take into account the three-dimensionality of the net structure or the fouling. The amount of fouling on nets can be estimated by subtracting the solidity of the clean net, or by calculating measures like the percentage net occlusion [15]. However, when net apertures are strongly occluded, more fouling might accumulate without being detected by the optical method. Other measures for the amount of net fouling, like wet weight or cumulative volume, do capture the total amount of fouling on a net, but such quantification measures to not deliver information about the fouling distribution on nets. Blockage of the flow through net openings has, in most cases, a much larger impact on the drag than skin friction on parts of fouling that is situated downstream from net strands and other fouling. Therefore, many traditional quantification measures for fouling seem less suited to be correlated with net drag. Whilesolidity does not measure the total amount of fouling, it is related to the blockage of net openings, which in turn is associated with drag. That makes solidity a good measure for the estimation of the effect of fouling on drag, which is supported by relatively small scatter between the exponential fits and measured data (see Fig 8).

### Clean nets

A number of studies have investigated the drag on clean nets and several authors give formulations for the drag based on net solidity, given uniform flow that is perpendicular to the net (e.g. [19], [20], [21], [22], [23]). Clean net data of the present study fit well to these results (see Fig 5). The present data is particularly well described by the cubic drag formulation by Aarsnes (1990) [19]. It fits equally well to results based on the formulation by Løland (1991) [20], but only within the range that the latter is validated for. At the highest solidities (Sn > 0.4) the measured drag was higher than predicted by this formulation. A good fit of the present data to previously developed formulations describing the relationship between solidity and drag indicate that the measurement setup was appropriate for this study. Even though the present data fit very well to existing cubic formulation, it was described even better with an exponential function.

The flow structure through net panels varies between nets (see Figs 5 and 6). Several factors may affect the flow around twines and knots, including the net solidity, the ratio of twine thickness to aperture size and the 3-dimensional structure of twines. The magnitude of the influence of such factors on the drag on nets is not certain, but the flow structure past twines will impact drag. While previous studies showed that net solidity as a single factor for the prediction of drag forces on nets may be sufficient (e.g. [19], [20], [21], [22], [23]), variations in the flow structure through nets (see Figs 5 and 6) may contribute to scatter in the relationship between solidity and drag. A number of authors suggested formulations for the drag coefficient of clean nets based on laboratory experiments (e.g. [19], [20], [21], [22], [23]). Differences between their formulations might at least partly be due to differences between the nets the authors used. An additional factor, describing the 3-dimensional structure of nets and twines, may allow an improvement of currently available drag formulations for nets.

### Comparison between clean and fouled nets

Similarly to clean net data, exponential functions describe the relationship of drag force and solidity reasonably well for both fouled net types, even though there is some scatter. Fig 8 shows clean and fouled net data with the drag force normalized to the net that experiences the highest drag. The data sets describing the relationship between solidity of and drag force on clean and fouled nets are statistically likely to be from distributions with different mean values (F_2.297_ = 202.88; p << 0.05). That means the data sets for clean and fouled nets can be treated independently from each other. Also the inclination of linear fits to log-normal transformations of the data sets is different for clean nets and fouled nets (F_2.297_ = 749.75; p << 0.05), and the increase in drag caused by changing the solidity of clean nets is much greater than that caused by increasing the solidity by adding fouling.

Starting with a fouled net panel and then reducing the amount of fouling will decrease the drag force on the net. When approaching a no-fouling situation the forces on the net should be identical to the forces on a clean net with the same solidity. Therefore, we expect that the functions describing drag on the fouled nets (types N1 and N2) intersect with a similar function for clean nets at the clean net solidity of net types N1 and N2. The functions describing fits to the clean net and fouled net data are therefore expected to intercept at Sn = 0.2 and Sn = 0.29 for fouled nets of type N1 and N2 respectively. The intercept was at or close to these solidities (0.2 for (N1) and 0.28 for (N2)), suggesting that the exponential fits to all data sets describe the relationship between solidity and drag very well, independently of whether solidity increase was due to clean net parameters or hydroid-fouling.

Plotting the data on a logarithmic y-axis (Fig 8b) makes exponential fits to the data appear linear, and from the figure differences in the increase of drag force on nets with increasing solidity becomes apparent. The figure visualizes the statistical result that the inclination of the best exponential fit to the clean net data differs significantly from those to the fouled net data sets. Drag increases faster for a given solidity increase for clean nets than when the solidity increase is due to fouling of the hydroid type. There is a statistical difference also between the inclinations of the solidity-drag relationship of the two fouled net types N1 and N2, but the actual difference in drag increase between those net types due to the same amount of fouling is very small. The drag increase on these nets for fouling-related solidity increase by 0.3 differs by little over 10%, while the difference to clean nets is > 40% (clean nets to fouled nets N1: solidity increase from 0.2 to 0.5; clean nets to fouled nets N2: solidity increase from 0.29 to 0.59). It seems likely that the relationship between hydroid-fouling related solidity increase and drag is similar to that for the two net types N1 and N2 within a solidity range between these types of net (solidity from 0.2 to 0.29). The amount of data in this study is limited and even though the correlation between solidity and drag is very good for all data sets, there is some scatter. Considering the small differences in the relationship between solidity and drag for the two fouled net types, the effect of hydroid-fouling on most nets within a solidity range from about 0.2 to 0.3 may be described adequately with either of the exponential functions describing the two fouled net data sets or with a function that gives an inclination of the log-normal transferred data that is in between the inclinations of the fits to N1 and N2 fouled net data.

If more fouled net data on this type of fouling was collected, thus improving the precision of the best fits to the fouled net data, the inclination could be estimated by making use of the proximity of a given net to the solidity of nets N1 or N2 (or other net types tested). An average of the exponents of the exponential fits for the two fouled net types could be chosen. With the log-normal transformed expression for the best fits for fouled nets of types A and B being Y_a_ = A_a_+b_a_·x and Y_b_ = A_b_+b_b_·x, parameter b_c_ of a similar expression for a net type C (with a solidity in between the net types A and B) could be scaled between b_a_ and b_b_ in dependence of the proximity of the clean net solidity between the net types. Scaling could be done linearly along the inclination of the fit to clean net data to account for an exponential relationship between solidity and drag for clean nets.

## Application

Regulations such as the Norwegian Standard NS9415 [8] acknowledge the importance of biofouling when considering the forces on aquaculture constructions. This standard asks for a solidity increase with 50% to account for fouling, but it does not give more detailed information about the effect of fouling or different fouling types. Having means to quantify the effect of fouling on the forces on net cages will allow for better risk analysis and the design and dimensioning of structural elements of aquaculture structures. Numerical models exist that describe forces and the behavior of aquaculture net cages in currents. In such models nets are often described in terms of clean net parameters like the diameter of net strands (see e.g. [24]). The database proposed in this study will allow the identification of transfer functions between fouled and clean nets that allow the parameterization of fouling on nets so that numerical models designed for clean nets can be used to model fouling.

**Fig 9:**
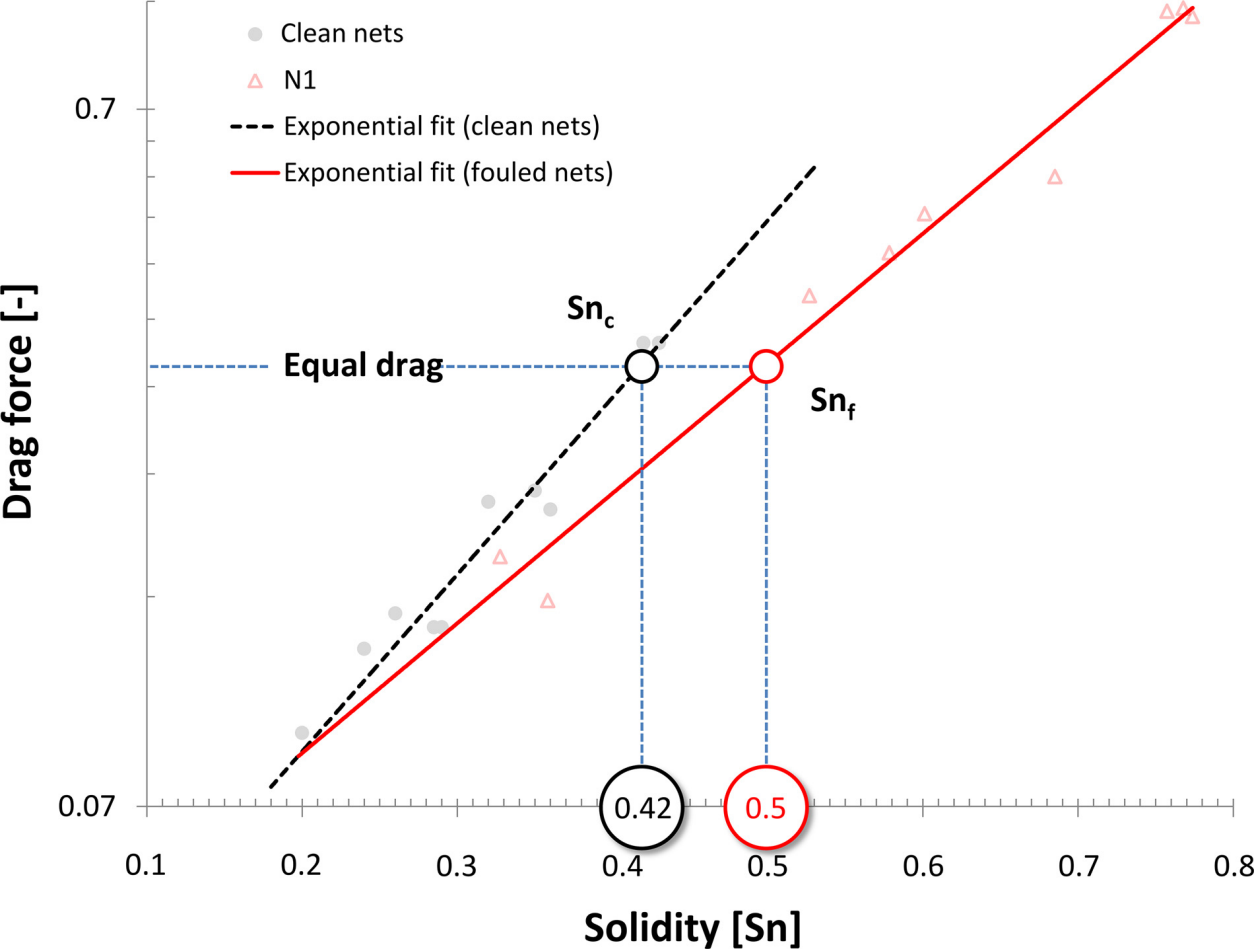
Exponential fits to the log-normal transformation of data shown in Fig 8. Note the logarithmic y-axis. The slope of the fit to fouled nets differs from the slope of the fit describing the drag increase of clean nets with increasing solidity. Knowing the initial clean net solidity and the present solidity of fouled nets (Sn_f_) allows a comparison between the drag on clean and fouled nets. In other words, a corresponding clean net solidity (Sn_c_) can be found that leads to similar drag forces on the net.

With regard to the forces on net panels and cages, which are key parameters for the design and behavior of aquaculture cage structures, the results of the present study may be used to determine what clean net solidity gives equivalent drag to a net with hydroid-fouling. This will then allow the use of existing models that predict the forces and behavior of clean nets (e.g. [24]) to be applied to fouled nets. An example of this parameterization is demonstrated in Fig 9. Here, the relationship between drag and solidity is plotted for clean nets (black) fouled nets of type N1 (red). After fouling accumulates, the solidity (as measured by image analysis) has increased to S_nf_ = 0.5. As shown in Fig 9, a clean net with solidity of S_nc_ = 0.42 has the same drag. Drag is associated with the water blockage and thus, nets that experience similar drag will result in similar water exchange. Therefore, the fouling may be parameterized by increasing the solidity in the clean-net model to 0.42.

As stated previously, this study considered only the effects of fouling by hydroids on nets that had initial solidities of 0.20 and 0.29, which represent the range of net solidities that currently are commonly used in Norwegian salmon aquaculture. Further experiments would be required to determine if the relationship between fouling and drag differs for net panels with initially clean solidities outside this range, if net type has an influence on fouling-induced drag, and most significantly the effect of different fouling types. The solidity of clean single nets used in this study ranged from 0.18 to 0.43, while some fouled nets approached solidities of 0.8. The drag on the heavily fouled nets was also higher than drag measured on any of the clean nets. Consequently, experiments on clean nets should also be extended to include higher solidities.

## Relevance of the Fouling Types

Previous studies measuring the drag on fouled nets did not distinguish between different fouling types, but the separation of fouling types for a more detailed analysis of the effect of fouling on the forces on nets has been called for [4]. Such information is valuable for several reasons. Fouling on nets was identified to have negative impacts on aquaculture structures. Fouling can add weight, change the behavior of net cages in the sea and it can influence the flow past net cages and the water exchange through nets (e.g. [2], [4], [25], [26], [27]). The present data is only on nets fouled with mainly hydroids and the hydranths were removed before drag tests. Hydroids are relatively small, flexible organisms and other fouling organisms are likely to influence the flow through nets differently. More rigid forms of fouling such as mussels are less likely to deform with the flow, and also have sharper corners that may promote the production of turbulence [28]. Thus, the relationship between drag force and solidity is expected to differ between fouling types. Similar tests with other fouling organisms can be used to build a more extensive database on the effect of fouling on drag on nets.

The results presented in this study are valid for hydroids without hydranths, which may be present on Norwegian net cages, as hydroids may shed and replace hydranths and as the bulk of hydroids on aquaculture nets may lose their hydranths while the hydrocauli stay intact (see also Fig 1b). Other fouling types, such as filamentous macroalgae and arborescent bryozoans, may behave similarly in currents and thus the results from this study are not restricted to this specific fouling, but may serve as a rough estimate for the effect of small, filamentous, flexible fouling organisms on net drag.

## Conclusions

This study shows that increasing the solidity of a net through fouling with hydroid hydrocauli produces a different (smaller) drag increase than increasing the solidity of a clean net by the same amount. Furthermore, the solidity of the initially clean net did not to a large degree influence the effect of hydroid fouling accumulation on the drag force. We therefore introduce only minor errors by applying the same function to parameterize drag increases from hydroid-type fouling for net panels that have clean (no fouling) solidities between 0.2 and 0.29, which covers the range of nets typically used in Norwegian salmon farming.

A method of parameterizing the effects of fouling in terms of an equivalent increase in clean net solidity is demonstrated, using the drag on nets as a proxy for the effect of fouling on the flow past nets. Measurements were conducted for one type of fouling (hydroids: small, flexible, filamentous organisms), and similar measurements should be carried out on nets with other types of fouling to build a database including the most important fouling organisms on aquaculture nets. Such information will help to implement the effect of fouling into numerical models that were developed for clean nets and therefore be used in the design and evaluation of the performance of aquaculture structures.

Fouling on nets can quickly increase solidity and there is very little data on highly solid clean nets that would allow a valid comparison of the drag on clean and fouled nets. Therefore, nets with very high solidity should be included into measurements to validate or improve available models for higher net solidities. This is especially interesting, as the flow mechanisms may change with varying net solidity. At low solidities single twines may evoke separate wakes, while the flow through nets with high solidities may be characterized by jet-flow through net apertures [25].
